# A case of variant of GBS with positive serum ganglioside GD3 IgG antibody

**DOI:** 10.1186/s13052-024-01682-1

**Published:** 2024-06-03

**Authors:** Jiao Xue, Zhenfeng Song, Hongshan Zhao, Zhi Yi, Fei Li, Chengqing Yang, Kaixuan Liu, Ying Zhang

**Affiliations:** 1 Department of Pediatric Neurology, The Affiliated Hospital of Qingdao University, No. 1677 Wutaishan Road, Qingdao, Shandong 266000 China; 2 Department of Anesthesiology, the Affiliated Hospital of Qingdao University, No. 1677 Wutaishan Road, Qingdao, Shandong 266000 China

**Keywords:** Guillain-Barré syndrome, Acute bulbar palsy-plus syndrome, Facial paralysis, Ganglioside GD3 antibody

## Abstract

**Background:**

Acute bulbar palsy-plus (ABPp) syndrome is an unusual variant of Guillain-Barré syndrome (GBS). Anti-GT1a and anti-GQ1b antibodies have been reported in patients with ABPp, but without reports related to GD3 antibodies.

**Methods:**

Clinical data of a patient diagnosed as ABPp syndrome were reviewed clinically. And we summarized the GBS patients with ABP and facial paralysis reported in the literature.

**Results:**

We reported a 13-year-old girl presented with asymmetric bifacial weakness, bulbar palsy and transient limb numbness, and had positive serum IgG anti-GD3 antibody. Through reviewing the GBS patients with ABP and facial paralysis reported previously, we found that facial palsy could be unilateral or bilateral. The bilateral facial palsy could present successively or simultaneously, and could be symmetrical or asymmetrical. Other common symptoms included ophthalmoplegia, sensory abnormality and ataxia. IgG anti-GT1a and IgG anti-GQ1b antibodies were the most frequent. Most of the patients had full recovery within two weeks to one year of follow-up.

**Conclusions:**

We reported a patient with asymmetric bifacial palsy and bulbar palsy, which seemed to fit the diagnosis of ABPp syndrome. This was the first report of ABPp variant of GBS with positive serum ganglioside GD3 IgG antibody.

**Supplementary Information:**

The online version contains supplementary material available at 10.1186/s13052-024-01682-1.

## Background

Guillain-Barré syndrome (GBS) is an immune-mediated polyradiculoneuropathy, which is subclassified into classic and localized forms [1]. More rare variants include the bifacial weakness with paresthesias and acute bulbar palsy-plus (ABPp) syndrome [2, 3]. The former is characterized by isolated bifacial weakness and distal limb paresthesias [2], and the latter presents with multiple cranial neuropathies without neck or limb weakness [3]. Some specific anti-ganglioside antibodies were closely related to the clinical features of classic GBS and its variants. A comparative study by Ito et al. [4] revealed that anti-GQ1b antibodies were present in 83% of patients with Miller Fisher syndrome (MFS) and 68% of patients with Bickerstaff brainstem encephalitis (BBE). Half of patients with pharyngeal-cervical-brachial (PCB) carried IgG anti-GT1a antibodies which might cross-react with GQ1b [5]. For patients with ABPp, the association with anti-GT1a and anti-GQ1b antibodies had been reported [3]. Here, we reported a 13-year-old girl who presented with asymmetric bifacial weakness, bulbar palsy and transient limb numbness, which was the first report of ABPp variant of GBS with positive serum ganglioside GD3 IgG antibody.

## Methods

The patient was admitted to our department in October 2022. Clinical data were reviewed clinically to obtain information. Blood cell count, blood biochemistry, blood electrolytes, blood ammonia, cytokine assay, cerebrospinal fluid (CSF) examinations, brain magnetic resonance imaging (MRI), magnetic resonance angiographyscans (MRA), magnetic resonance venography (MRV) and electromyography were performed. Serum and CSF ganglioside IgM and IgG antibodies were determined using BLOT.

## Results

A 13-year-old normally developing girl presented to our hospital with worsening facial weakness. The patient presented with incomplete closure of the left eye and deviated mouth to right, with hypogeusia, since 1.5 months ago. She was presumed as Bell’s palsy by the local pediatrician and was treated with traditional Chinese medicine and acupuncture therapy for 3 weeks. Her left facial weakness improved slightly. While 3 days prior to presentation, she presented with new symptoms that incomplete closure of the right eye, deviated mouth to left, numbness of the tongue, earache, hypogeusia, dysphagia and paroxysmal weakness and numbness of the right upper limb.

On admission, she appeared alert and oriented. Nervous system physical examination revealed flat and few expression of her face, incomplete closure of the bilateral eyes, effacement of nasolabial fold and forehead wrinkle, more pronounced on the right side, and deviated mouth to left. She was noted to have lower tone voice with slightly nasal intonation. She could not swallow properly and had dysphagia to solids. There was bilateral paralysis of the soft palate and loss of pharyngeal reflex. Her muscle strength and tension was normal. The deep tendon reflexes were elicited symmetrically. Pathological reflex examination was negative. Examinations of coordinate movement including Romberg test, finger-to-nose, alternating movement and heel-to-shin tests were normal.

Laboratory test results indicated that routine blood, liver and kidney function, electrolytes, erythrocyte sedimentation rate and antinuclear antibodies were normal. CSF results showed normal white cells (2 × 10^6^/L), protein (351.6 mg/L; reference 120–600 mg/L) and normal level of immunoglobulin (Ig) including IgG (25.8 mg/L), IgA (2.05 mg/L), IgM (0.56 mg/L) and albumin (145 mg/L). Brain MRI, MRA and MRV were normal. Electromyography of bilateral upper limbs and facial muscles showed neurogenic damage of bilateral facial nerves (Table 1). Serum ganglioside GD3 IgG antibody was positive, and CSF ganglioside IgM and IgG antibodies (GD1a, GD1b, GD2, GD3, GM1, GM2, GM3, GM4, GT1a, GT1b, GQ1b, Sulfatide) were negative. She was diagnosed as GBS, most consistent with the ABPp variant. Intravenous immunoglobulin (IVIG; 2 g/kg) was given within five consecutive days and mild improvement was noted in her facial weakness. Two weeks after discharge, her swallowing function improved without obvious dysphagia, and facial expressions increased significantly.

**Table 1:** Nerve conduction study

Motor nerves	Teminal latency (ms)	Amplitude (µV)	Nerve conduction velocity (m/s)	F waves
Median (left/right)	2.7/2.1, N (≤ 4.4)	7.5/6.1, N (≥ 4.0)	73.3/61.7, N (≥ 49.0)	Normal
Ulnar (left/right)	2.1/2.4, N (≤ 3.3)	7.4/7.2, N (≥ 6.0)	67.5/70.2, N (≥ 49.0)	Normal
Tibial (left)	3.9, N (≤ 5.8)	16.5, N (≥ 4.0)	47.5, N (≥ 41.0)	Normal
Peroneal (left)	3.4, N (≤ 6.5)	5.3, N (≥ 2.0)	51.5, N (≥ 44.0)	Normal
Facial-orbicularis oculi (left/right)	0.9/3.6, N (≤ 3.1)	1.0/1.4, N (≥ 1.0)		
Facial-orbicularis oris (left/right)	1.1/1.4, N (≤ 4.2)	0.9/1.2, N (≥ 1.0)		
Facial-frontalis (left/right)	3.1/3.7, N (≤ 3.5)	0.6/0.4, N (≥ 1.0)		
**Sensory nerves**	**Teminal latency (ms)**	**Amplitude (µV)**	**Nerve conduction velocity (m/s)**	
Median (left/right)	1.5/1.2, N (≤ 3.5)	119.5/85.3, N (≥ 20.0)	66.6/81.9, N (≥ 50.0)	
Ulnar (left/right)	1.6/1.3, N (≤ 3.1)	71.3/130.9, N (≥ 17.0)	64.1/84.6, N (≥ 50.0)	
Sural (left)	3.0, N (≤ 4.4)	9.6, N (≥ 6.0)	46.6, N (≥ 40.0)	
Superficial peroneal (left)	3.0, N (≤ 4.4)	16, N (≥ 6.0)	61.6, N (≥ 40.0)	
**Blink reflex latency (ms)**	**R1 latency Ipsiateral**	**R2 latency Ipsiateral**	**R2 latency Contralateral**	
Left	7.9, N (≤ 13)	33.8, N (≤ 41)	Fail to get	
Right	Fail to get	Fail to get	26.6, N (≤ 44)	

N() – normal value

## Discussion

GBS is an immune-mediated inflammatory disease of peripheral nervous system [6]. The incidence is approximately 1–2 per 100,000 person [6]. Some distinct clinical variants of GBS included weakness limited to the cranial nerves (ABPp syndrome, bilateral facial palsy with paraesthesias), lower limbs (paraparetic variant), upper limbs (pharyngeal-cervical-brachial weakness) and MFS [1, 7, 8]. Here, we reported a 13-year-old girl who presented with asymmetric bifacial weakness, bulbar palsy, transient limb numbness and had positive serum ganglioside GD3 IgG antibody, which was the first case of ABPp reported with positive GD3 IgG antibody.

ABPp syndrome, as a rare variant of GBS, characterized with ABP plus other cranial symptoms or additional signs (such as ataxia) but without neck or limb weakness [3, 9]. The glossopharynx and vagus nerves are adjacent to each other and are often damaged at the same time, presenting as hoarseness, dysphagia, drinking cough and pharyngeal reflex disappear, called bulbar paralysis (true bulbar paralysis). Kim et al. [3] and Cao et al. [9] proposed the diagnostic criteria of ABPp syndrome as follows: (1) prominent ABP, absence of neck and limb weakness; (2) other cranial involvements or gait ataxia or both; (3) compliance with the illness pattern of general GBS; and (4) absence of identified alternative diagnosis. According to the presentation of our patient here, we summarized the GBS patients with ABP and facial paralysis reported previously, which fit the diagnosis of ABPp syndrome (Table 2) [3, 10–23]. The literature search showed twenty-three patients previously, eighteen adults (age: 20y-67y) and five children (age: 10 m-13y). Twelve of the 24 cases (including our case) were female. The facial palsy was unilateral in 12 patients and bilateral in 10 patients. The bilateral facial palsy could present successively or simultaneously, and could be symmetrical or asymmetrical. Other symptoms included ophthalmoplegia (including ptosis and diplopia, 16/24), sensory abnormality (12/24), ataxia (9/24), neck weakness (1/24) and limb weakness (1/24). It suggested that ABPp syndrome were rarely ‘pure’, which could manifest features that were typical of other variant forms, such as bifacial weakness with paresthesias, MFS or PCB variant, but it differs from them in the prominent bulbar palsy. Deep tendon reflexes were absent or decreased in 14 patients and remained normal in 10 patients. CSF albuminocytological dissociation was found in 68.4% patients (13/19 cases available).

**Table 2:** Summary of previously reported GBS patients with acute bulbar palsy and facial paralysis

No.	Age	Sex	Bulbar palsy	Facial palsy	Initial symptoms	Other symptoms	Deep tendon reflexes	CSF albuminocytological dissociation	antiganglioside antibodies	Treatment	Follow-up
Our case	13y	F	√	asymmetric bifacial	facial weakness	transient limb numbness	Normal	No	IgG anti-GD3	IVIG	remarkably improved after 3 weeks
Dukkipati et al. [10]	3y	F	√	asymmetric bifacial	facial weakness	dysphagia, dysphonia, ataxia	Absent	Yes	Negative	IVIG	Recovery on three-month follow-up
Rojas-Garcia et al. [11].	54y	M	√	bifacial	dysarthria, dysphagia	masticatory muscle and tongue weakness	Normal	Yes	IgG anti-GM3, GD1a, GT1b	IVIG	Mild improvement after 1 year
Yadav et al. [12]	10 m	M	√	unilateral	facial deviation, dysphagia	nasal intonation	Decreased	NA	NA	Support treatment	recovery after 4 months
Sharma et al. [13]	5y	M	√	unilateral	limb pain, difficulty walking	ataxia	Absent	Yes	NA	IVIG	remarkably improved after 2 weeks
Ray et al. [14]	13y	F	√	unilateral	facial deviation, dysphagia	nasal intonation, areflexia	Absent	NA	NA	physiotherapy	Improvement within next 4 weeks
Onodera et al. [15]	29y	M	√	bifacial	difficulty in speaking and swallowing	mild neck weakness	Normal	Yes	IgG anti-GT1a	plasmapheresis	recovery after one month
Yu et al. [16]	48y	M	√	bilateral symmetrical	bilateral ptosis	diplopia, dysarthria, dysphagia	Normal	Yes	IgG anti-GQ1b	IVIG	mild diplopia, dysphonia, dysphagia after 11 weeks
Tan et al. [17]	57y	F	√	unilateral	altered sensation of extremities, ptosis, diplopia, slurred speech, difficulty swallowing	limb weakness, respiratory muscle weakness	Absent	Yes	IgG anti-GQ1b, GT1a	plasma exchange	facial weakness resolved completely by day 43, ophthalmoplegia improve on day 40, ambulate with a walking frame on day 44
Kim et al. [3]	20y	F	√	unilateral	gait ataxia	external ophthalmoplegia, areflexia	Absent	No (day 2)	IgG anti-GT1a, GQ1b	IVIG	full recovery after 6 weeks
Kim et al. [3]	54y	F	√	unilateral	dysarthria	external ophthalmoplegia, areflexia, sensory abnormality, gait ataxia	Absent	No (day 2)	IgG anti-GT1a, GQ1b, IgM anti-GT1a, GQ1b	IVIG	full recovery after 7 weeks
Kim et al. [3]	26y	F	√	unilateral	diplopia	external ophthalmoplegia, areflexia, sensory abnormality, gait ataxia	Absent	No (day 2)	IgG anti-GT1a, IgM anti-GT1a	IVIG	full recovery after 16 weeks
Kim et al. [3]	21y	M	√	unilateral	dysarthria, dysphagia	external ophthalmoplegia, areflexia,	Absent	Not determined	IgG anti-GT1a, GQ1b	IVIG	full recovery after 7 weeks
Kim et al. [3]	27y	M	√	unilateral	diplopia	external/internal ophthalmoplegia, areflexia, sensory abnormality, gait ataxia	Absent	Yes	IgG anti-GT1a	IVIG	not fully recovered after 30 weeks
Kim et al. [3]	20y	M	√	unilateral	numbness of limbs	external ophthalmoplegia, areflexia, sensory abnormality, gait ataxia	Absent	No (day 2)	IgG anti-GQ1b, GT1a, GM2	IVIG	full recovery after 4 weeks
Edvardsson et al. [18]	54y	M	√	bilateral	diplopia	external ophthalmoplegia	Normal	Yes	IgG anti-GQ1b	IVIG	full recovery after 8 weeks
Pavone et al. [19]	10y	M	√	bilateral symmetrical	left ptosis, diplopia, dysarthria	external/internal ophthalmoplegia, masticatory muscle and tongue weakness	Normal	No	Negative	Steroids, IVIG	full recovery after 8 weeks
Lyu et al. [20]	67y	F	√	unilateral	diplopia, numbness of limbs	external ophthalmoplegia, sensory abnormality	Normal	Yes	Not determined	None	full recovery after 3 weeks
Lyu et al. [20]	33y	M	√	bilateral	diplopia, facial palsy, dysarthria	external ophthalmoplegia,	Normal	Yes	Not determined	IVIG	full recovery after 6 weeks
Lyu et al. [20]	47y	M	√	bilateral	unilateral facial palsy, slurred speech, a tendency to choke	external ophthalmoplegia, sensory abnormality	Normal	Yes	IgG anti-GQ1b	None	completely recovered within 1 year
Kamakura et al. [21]	23y	F	√	unilateral	numbness of limbs	sensory abnormality, gait ataxia	Normal	Yes	IgG anti-GQ1b, GT1a	plasma exchange	full recovery (exact time unknown)
Koga et al. [22]	34y	F	√	NA	dysarthria, dysphagia, numbness of limbs	external ophthalmoplegia, areflexia, sensory abnormality	Absent	Not determined	IgG anti-GM1b, GT1a, IgM anti-GM1b, GT1a	plasma exchange	mild ophthalmoplegia (5 months)
Koga et al. [22]	29y	F	√	NA	dysarthria, dysphagia, numbness of limbs	external ophthalmoplegia, areflexia, sensory abnormality	Absent	Not determined	IgG anti-GT1a, GQ1b, GM1b, IgM anti-GM1b, GT1a, GQ1b, GalNAc-GD1a	None	mild ophthalmoplegia (4 weeks)
Banerji et al. [23]	41y	F	√	bilateral	dysarthria, dysphagia, gait ataxia	external ophthalmoplegia, areflexia	Absent	Yes	Not determined	None	full recovery after 2 weeks

F: female; M: male; IVIG: intravenous immunoglobulin; NA: not available

Moreover, eighteen patients of the 23 cases (Table 2) [3, 10–23] underwent serological assay, and antiganglioside antibodies were identified in 88.9% patients (16/18 cases). IgG anti-GT1a antibody (11, 68.8%) was the most frequent, followed by IgG anti-GQ1b (10/16, 62.5%), IgM anti-GT1a (4/16), IgM anti-GQ1b (2/16), IgG anti-GM1b (2/16), IgM anti-GM1b (2/16), IgG anti-GM2 (1/16), IgG anti-GM3 (1/16), IgG anti-GD1a (1/16), IgM anti-GD1a (1/16), IgG anti-GT1b(1/16). Here, we reported the first patient with facial palsy and bulbar palsy that had positive IgG anti-GD3 antibody.

Gangliosides are sialic acid-containing glycosphingolipids (GSLs) ubiquitously distributed in tissues and body fluids, and are more abundantly expressed in the nervous system [24]. The expression levels and patterns of gangliosides undergo dramatic changes during brain development [25]. In the early embryonic brain, the pattern of ganglioside expression is characterized by the abundance of simple gangliosides, such as GM3 and GD3. As the brain develops, the expression of these simple gangliosides is down-regulated with concomitant up-regulation of complex gangliosides such as GM1, GD1a, GD1b, and GT1b, etc [26]. Thus, GD3, GD-1b, GT-1a, and GQ-1b are structurally similar. To some extent, this dynamic correlation might explain the similar symptoms between our patient with positive anti-GD3 antibody and other ABPp with GT-1a or GQ-1b antibodies. Through autoimmune reactions, GD3 antibodies might bind to gangliosides on the surface of nerve cell membranes, promoting neurological damage [27].

IVIG and plasma exchange, as the first-line treatment of GBS, were used in most patients (14/24 cases and 4/24 cases, respectively) with good effects. In addition, six of the 24 patients had spontaneous improvement under symptomatic and supportive treatment [12, 14, 20, 22, 23]. Most of the patients (15/24 cases) had full recovery within two weeks to one year of follow-up.

## Conclusions

In conclusion, we reported a patient with asymmetric bifacial palsy and bulbar palsy, which seemed to fit the diagnosis of ABPp variant. This was the first report of ABPp variant of GBS with positive serum ganglioside GD3 IgG antibody.

### Electronic supplementary material

Below is the link to the electronic supplementary material.

Supplementary Material 1

## Data Availability

The datasets generated and analyzed during the current study are all shown in the manuscript.

## Abbreviations

GBS: Guillain-Barré syndrome
ABPp: acute bulbar palsy-plus
MFS: Miller Fisher syndrome
BBE: Bickerstaff brainstem encephalitis
PCB: pharyngeal-cervical-brachial
CSF: Cerebrospinal fluid
Ig: immunoglobulin
MRI: magnetic resonance imaging
MRA: magnetic resonance angiographyscans
MRV: magnetic resonance venography
IVIG: intravenous immunoglobulin
GSLs: acid-containing glycosphingolipids
