# Supplementary material for: A case of variant of GBS with positive serum ganglioside GD3 IgG antibody
Source: Ital J Pediatr. 2024 Jun 3;50:109. doi: 10.1186/s13052-024-01682-1 (PMC11149178; doi:10.1186/s13052-024-01682-1)
Supplement: Supplementary file 1 — Supplementary Material 1 [file 13052_2024_1682_MOESM1_ESM.doc]

Dear Editors and Reviewers:

Thank you for your letter and for the reviewers’ comments concerning our manuscript entitled “Efficacy of levetiracetam in electrical status epilepticus during sleep of children: a multicenter experience.” (manuscript number: PNU-D-13-00455). Your comments are all valuable and very helpful to the revision and improvement of our paper. In addition, your feedback served as a guide in improving the significance of our research. We have studied your comments carefully and have made corrections that we hope meet your approval. Revised portions are marked in red. Below are the main corrections we implemented and our responses to the reviewers’ comments. If it still does not meet the requirements, we are willing to make further modifications.

With best regards,

Ying Zhang

The below is the reply to each point which is being raised by the reviewers.

Reviewer(s)' Comments to Author:

**Reviewer #1:**

1. The novelty of this article is that the patient is a Variant of Guillain- Barré Syndrome with positive serum ganglioside GD3 IgG antibody.Suggest changing the title： A case of Variant of GBS with Positive Serum Ganglioside GD3 IgG antibody.

Answer: Thank you very much for your suggestion, we have changed the title as: A case of Variant of GBS with Positive Serum Ganglioside GD3 IgG antibody.

2. The introduction and discussion section of the article should focus on neuroinflammation (an acute bulbar palsy plus variant of Guillain- Barré syndrome) caused by ganglioside GD3 IgG antibodies. During the acute phase of Zika virus infection, the level of GD3 autoantibodies will significantly increase. Through autoimmune reactions, GD3 antibodies may bind to gangliosides on the surface of nerve cell membranes during Zika virus infection, promoting neurological damage(Koike H. Zika virus and Guillain Barr é syndrome. Brain Nerve, 2018, 70 (2): 113-120).

Answer: I have revised the introduction and discussion section and added contents about neuroinflammation caused by ganglioside GD3 IgG antibodies. The details are described below: we added “Some specific anti-ganglioside antibodies were closely related to the clinical features of classic GBS and its variants. A comparative study by Ito et al. revealed that anti-GQ1b antibodies were present in 83% of patients with Miller Fisher syndrome (MFS) and 68% of patients with Bickerstaff brainstem encephalitis (BBE). Half of patients with pharyngeal-cervical-brachial (PCB) carried IgG anti-GT1a antibodies which might cross-react with GQ1b. For patients with ABPp, the association with anti-GT1a and anti-GQ1b antibodies had been reported” in the introduction section. We added “Gangliosides are sialic acid-containing glycosphingolipids (GSLs) ubiquitously distributed in tissues and body fluids, and are more abundantly expressed in the nervous system. The expression levels and patterns of gangliosides undergo dramatic changes during brain development. In the early embryonic brain, the pattern of ganglioside expression is characterized by the abundance of simple gangliosides, such as GM3 and GD3. As the brain develops, the expression of these simple gangliosides is down-regulated with concomitant up-regulation of complex gangliosides such as GM1, GD1a, GD1b, and GT1b, etc. Thus, GD3, GD-1b, GT-1a, and GQ-1b are structurally similar. To some extent, this dynamic correlation might explain the similar symptoms between our patient with positive anti-GD3 antibody and other ABPp with GT-1a or GQ-1b antibodies. Through autoimmune reactions, GD3 antibodies might bind to gangliosides on the surface of nerve cell membranes, promoting neurological damage.” in the discussion section.

3. It is suggested that the results of EMG and CSF GD3 IgG antibody should be supplemented in the case.

Answer: I have supplemented the results of EMG in the manuscript as Table 1. And added the results of CSF GD3 IgG antibody should be supplemented in the case report.

**Reviewer #2:**

Thanks for reviewing manuscript "Acute bulbar palsy-plus syndrome with bilateral facial paralysis in a Chinese child"

The main criticism is due to the definition lack of bulbar function in pediatric patient please in the discussion section add bulbar function and monitoring. Moreover in the clinical case report should evidente to see monitoring bulbar action for the patient.

Answer: I have supplemented the definition of bulbar function in the discussion section that “The glossopharynx and vagus nerves are adjacent to each other and are often damaged at the same time, presenting as hoarseness, dysphagia, drinking cough and pharyngeal reflex disappear, called bulbar paralysis (true bulbar paralysis)”. And in the clinical case report, we described the bulbar function and monitoring of the patient as follows: “She was noted to have lower tone voice with slightly nasal intonation. She could not swallow properly and had dysphagia to solids. There was bilateral paralysis of the soft palate and loss of pharyngeal reflex.” “Two weeks after discharge, her swallowing function improved without obvious dysphagia, and facial expressions increased significantly.” If it still does not meet the requirements, we are willing to make further modifications.
